# The Atomic Oxygen Erosion Resistance Effect and Mechanism of the Perhydropolysilazane-Derived SiOx Coating Used on Polymeric Materials in Space Environment

**DOI:** 10.3390/polym14020322

**Published:** 2022-01-13

**Authors:** Hong Qi, Qingshan Shi, Yuhai Qian, Yueming Li, Jingjun Xu, Caihong Xu, Zheng Zhang, Xiaobao Xie

**Affiliations:** 1 Guangdong Provincial Key Laboratory of Microbial Culture Collection and Application, Institute of Microbiology, Guangdong Academy of Sciences, Guangzhou 510070, China; hqi18s@163.com; 2 State Key Laboratory of Applied Microbiology Southern China, Institute of Microbiology, Guangdong Academy of Sciences, Guangzhou 510070, China; 3 Aluminum Valley Industrial Technology Institute, Zouping 256200, China; qianyuhai@iti-alvalley.cn; 4 School of Materials Science and Engineering, Dongguan University of Technology, Dongguan 523808, China; ymli11s@alum.imr.ac.cn; 5 Shenyang National Laboratory for Materials Science, Institute of Metals Research, Chinese Academy of Sciences, Shenyang 110016, China; jjxu@imr.ac.cn; 6 Beijing National Laboratory for Molecular Sciences (BNLMS), Institute of Chemistry, Chinese Academy of Sciences, Beijing 100190, China; caihong@iccas.ac.cn; 7 Guangdong Provincial Key Laboratory of Electronic Information Products Reliability Technology, Guangzhou 510610, China

**Keywords:** PHPS, silica coating, atomic oxygen, erosion-corrosion, resistance mechanism

## Abstract

In this work, the atomic oxygen (AO) erosion-resistance effect and mechanism of the Perhydropolysilazane (PHPS) coating were investigated from the perspective of element distribution in the depth direction. The results revealed that the coating demonstrated good adhesion and intrinsic AO erosion-resistance, which was attributed to the composition gradient formed in the coating. Moreover, the oxygen ratio of the SiOx on top layer of the coating could be elevated during AO exposure, strengthening the Ar ion etching durability of the coating. According to these results, an AO erosion-resistance mechanism model of the PHPS-derived SiOx coating was finally obtained.

## 1. Introduction

Polymeric materials, relying on their good mechanical, chemical, and dielectric properties, are widely used on spacecraft to carry the flexible equipment, package the spacecraft, or reflect the solar radiation and so on [1,2]. The low Earth orbit (LEO) is typically used for the operation of various satellites. However, in LEO, high-flux atomic oxygen (AO) and intense vacuum ultraviolet (VUV) are the most important factors for the erosion of polymeric materials of spacecrafts [3,4,5,6]. AO and VUV could cause serious damage to polymers, thereby decreasing the chemical stability and service life of the spacecraft. Such damage mainly occurs due to these three aspects. First, AO is the most abundant atmosphere composition in LEO, and with its density reaching about 10^7^–10^10^ atom cm^−3^, has about 8 km s^−1^ impacting velocity at ram impact velocities, corresponding to the total kinetic energy of 4.5–5 eV [7,8,9]. Second, AO exhibits two active points that can easily form chemical bonds with other atoms or molecules [10]. In addition, VUV with a wavelength of 110–200 nm can break chemical bonds [11]. The application of coating systems has been reported to be effective to overcome such issues [12,13,14]. Initially, an organic coating was deployed, such as polysiloxane, polysilazane, and fluorinated polymers. The organic coatings usually bond to the polymer material surface firmly and have better flexibility, However, when exposed to UV radiation in the LEO environment, they are prone to aging [15,16,17]. Then, the inorganic coating was applied. The inorganic coating was mainly achieved by applying an inert SiO_2_ or Al_2_O_3_ composition, or even directly adopting silica and alumina as coating materials [6,11,17,18,19,20,21,22,23]. However, the adhesion property of these coatings applied on flexible polymeric membrane and film are poor. The good AO erosion-resistance and good adhesion cannot be obtained simultaneously by the inorganic coating. In recent years, in order to solve this problem, gradient coating was proposed, which can improve the bonding performance of coatings by establishing a gradient structure and gradient composition, such as coatings prepared by LAD, plasma polymerization deposition, ion implantation (IIP), filter cathode vacuum arc (FCVA), and electron beam (E-beam) methods [18,23,24]. However, the instruments and procedures for preparing gradient coating are complex and expensive. Therefore, it is necessary to prepare a kind of inorganic coating with excellent adhesion to the flexible polymeric substrate, and at the same time the preparation process is simple, economical, and environmentally friendly.

In recent years, PHPS has attracted extensive attention. PHPS is a type of inorganic polymer comprised of a Si–N skeleton with Si–H side groups. It is extremely reactive with H_2_O and O_2_ due to the polarity of Si–N. Thereby PHPS could converting to SiO_x_ at low temperatures under moisture [25,26,27,28,29]. As a coating material, a layer of SiO_x_ could form in-situ on the top surface of the PHPS coating. This coating system exhibits intrinsic advantages, such as superior adhesion strength, transparency, and a self-healing ability. These characteristics can lead to the simple preparation of a ceramic coating and demonstrate promise for large-scale applications and in situ repair [30,31,32,33,34]. Furthermore, the low-temperature process is suitable for polymer substrates, which is attractive for preparing inorganic SiO_x_ coatings on polymers. Examples include a flexible resistive random access memory device on polyimide by a PHPS-derived SiO_x_ method [35], water-vapor adsorption/desorption performance improvement of polytetrafluoroethylene (PTFE) by a modified PHPS-derived amorphous silica coating [36], and a luminescence-enhanced, stable PHPS-derived nanopatterned CsPbBr_3_ perovskite nanocrystal (PNC)-SiO_2_ film at low temperatures (<200 °C) [37]. PHPS conversion and coating properties are affected by moisture, temperature, and catalyst [38,39,40,41]. Yang et al. [39] have reported that the Si–O–Si bond formation as well as the hardness and Young’s modulus of PHPS-derived coatings change at different temperatures.

A study reported previously by our group revealed that after hydrolyzing in water for 5 min at room temperature, a PHPS-derived SiO_x_ layer formed on the top surface of the coating and the coating demonstrated excellent AO resistance [22]. The previous study was mainly focused on the surface composition of the coating, and the depth distribution of the elements were not available. However, the distribution of elements in depth was crucial to reveal the initial AO erosion resistance effect of the coating and provide additional information about the conversion of materials exposed to AO [42]. Therefore, in this study, the AO erosion-resistance mechanism is determined by the comparison of the element distribution along the depth direction of the PHPS-derived SiO_x_ coating before and after AO exposure by XPS. In addition, the VUV irradiation resistance and adhesion strength of the PHPS-derived SiO_x_ coating were investigated before and after AO exposure. Finally, according to the results, an AO erosion-resistance mechanism model of the PHPS-derived SiO_x_ coating was proposed.

## 2. Materials and Methods

### 2.1. Coating Preparation

PHPS supplied by the Institute of Chemistry, Chinese Academy of Sciences, was used as the silica precursor. Its density, average molecular weight (M_n_), and polydispersity index (M_w_/M_n_) were 1.21 g cm^−3^, 900, and 3.4, respectively. Xylene (AR, 99%, Aladdin, Shanghai, China) was used as received. Commercial Kapton HN (polyimide film, 55-μm thickness) was selected as the substrate, which was activated according to the alkaline hydrolysis principle. The PHPS-derived SiO_x_ coating was prepared on Kapton by dip-coating method. First, the activated substrates were immersed in a solution of PHPS in xylene (5 wt.%) for 1 min and then pulled out at 500 μm s^−1^. Second, the sample was dried in clean air and subsequently treated at 220 °C in a furnace. Finally, the cooled samples were hydrolyzed with distilled water for 5 min. The thickness of the as-prepared PHPS-derived SiO_x_ coating was ~1 μm.

### 2.2. AO/VUV Exposure Test

AO/VUV exposure tests were conducted in a ground-based space environment simulation facility. Details of the construction and operation sequence of this facility have been reported elsewhere [43,44]. The AO/VUV exposure test was conducted in a vacuum chamber at 0.1 Pa and at an O_2_ flow rate of 9.0 SCCM. The height of the sample holder was fixed at 15 cm. The total AO fluence was controlled by the exposure time. A deuterium lamp with a wavelength of 115–400 nm and an intensity of 9.56 μW cm^−2^ (distance: 15 cm) was used as the VUV source.

The erosion yield coefficient (*E*_y_, cm^3^ atom^−1^) of a sample is a key parameter that reflects the AO resistance of a material. It is defined as the volume loss caused by the attack of one atomic oxygen, which can be calculated by Equation (1):

(1) $$E_{y}=\frac{\Delta M}{\rho \;A\;t\;f}\;\left({\mathrm{cm}}^{3}\;{\mathrm{atom}}^{-1}\right)$$

where *E*_y_ is the erosion yield (cm^3^ atom^−1^), Δ*M* is the weight loss of the sample (g), *A* is the exposed surface area (cm^2^), *ρ* is the density (g cm^−3^) of the sample (0.79 g cm^−3^), *t* is the exposure time (s), and *f* is the AO flux (2 × 10^16^ atoms cm^−2^ s^−1^).

Based on the weight change of the standard sample (Kapton) during the AO erosion test, the AO fluence can be determined from Equation (2):

(2) $$F=\frac{\Delta M_{K}}{\rho_{K}\;A\;E_{y}}$$

where Δ*M_k_* is the weight loss of Kapton (g), *ρ_k_* is the density (g cm^−3^) of Kapton (1.42 g cm^−3^), *A* is the exposed surface area (cm^2^), and *E_y_* of Kapton is 3 × 10^−^^24^ cm^3^ atom^−^^1^.

To obtain the erosion kinetics of the hybrid coating during AO/VUV exposure, the samples were alternately removed from the exposure chamber and weighed using an analytical balance (Sartorius, model BP211-D) with an accuracy of 10^−5^ g. Next, the samples were placed into the chamber again for the subsequent exposure test.

### 2.3. Sample Characterization

The surface morphologies of the as-prepared coating and Kapton substrate before and after AO exposure were observed by scanning electron microscopy (SEM, Supra 35, LEO, Oberkochen, Germany). The chemical composition of the sample surface was characterized by energy-dispersive X-ray (EDS) spectrometry built into the SEM system. The 3D morphologies of the sample were obtained by a white light interferometer 3D profilometer (UP-DUAL MODE, Rtec, B San Jose, CA, USA).

The elemental composition and lateral and depth distribution of elements on the sample surface were characterized by X-ray photoelectron spectroscopy (XPS, ESCALAB 250, Thermo VG, Waltham, MA, USA). A base pressure of better than 3 × 10^−9^ mbar during spectral acquisition was achieved by using a Ti sublimation pump. Monochromatic Al Kα radiation was used as the excitation source, with an accelerating voltage of 15 kV and an output power of 150 W. Survey and core-level spectra were recorded at scan rates of 20 eV s^−1^ and 2 eV s^−1^, respectively. The spectra were acquired in parallel. A charge neutralizer was used during XPS analysis. The survey spot was 500 μm, and the scanning step was 0.1 eV. For the depth distribution of elements, the samples were continuously sputtered by a 3 kV Ar ion beam with a current of 2 μA, the etching rate is about 10 nm s^−1^, and the high-resolution narrow scans of the elements were measured per 100 s by XPS. As XPS was performed using a defocused source, the collected data were an average value over the area range of ~2 mm × 2 mm. XPSPEAK (version 4.1) software was used for peak fitting.

## 3. Results and Discussion

### 3.1. Morphology and Chemical Composition Characteristics

**Figure 1:**
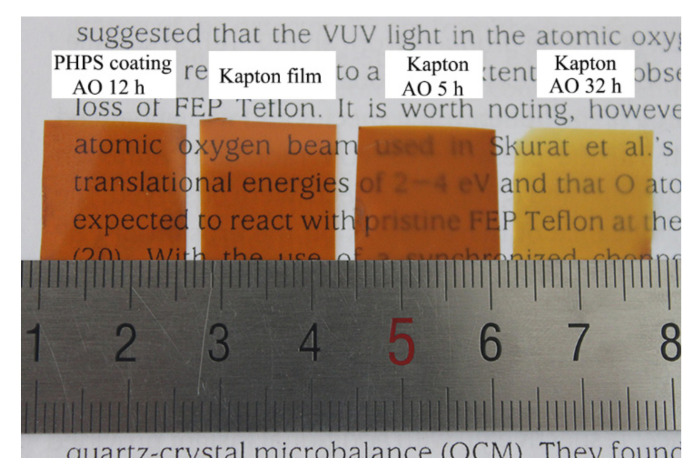
Digital photographs of the PHPS coating exposed to AO for 12 h, pristine Kapton film and Kapton exposed to AO for 5 h and 32 h.

Figure 1 shows the morphologies of the PHPS-coated Kapton eroded by AO for 12 h, pristine Kapton, and Kapton eroded by AO for 5 h and 32 h. Even after AO exposure for 12 h, the PHPS-coated Kapton film was still transparent and homogenous. Its transparency was comparable to that of the pristine Kapton film. On the contrary, pristine Kapton was visibly eroded by AO, and after exposure to AO for 5 h, its surface became frosted. At an exposure time of 32 h, the Kapton film got thinner. Its color faded and started to split into fragments. The significant contrast between the coated and uncoated Kapton reveals that the PHPS coating exhibits obvious AO resistance.

**Figure 2:**
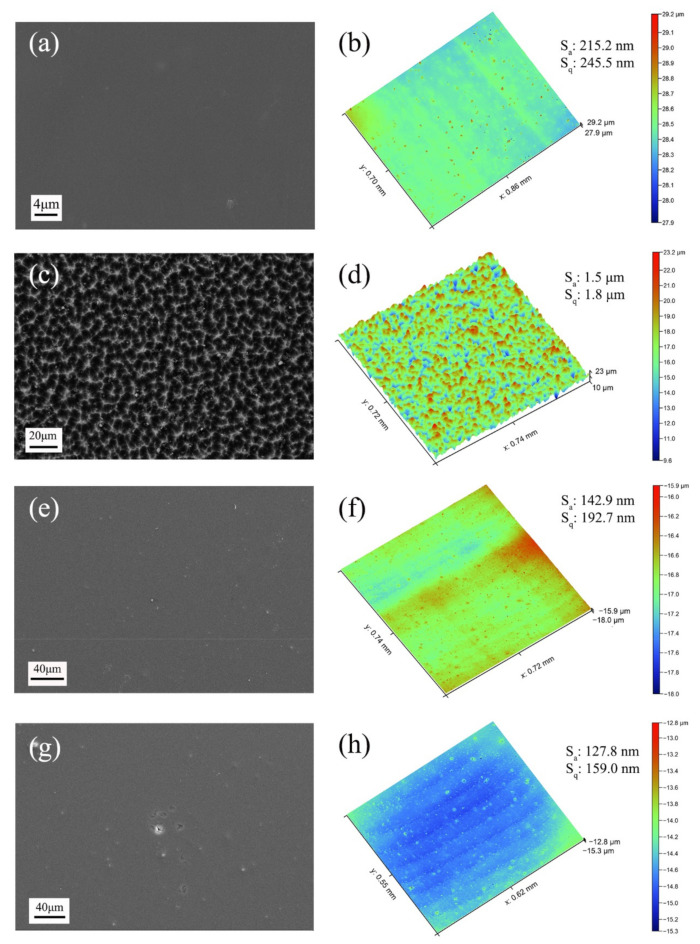
Surface morphologies of (**a**) Kapton, (**c**) Kapton after AO exposure, (**e**) as-prepared PHPS coating and (**g**) PHPS coating after AO exposure (Total fluence of AO exposure: 1.5 × 10^21^ atoms cm^−2^) and corresponding 3D morphologies of each sample were displayed aside in (**b**,**d**,**f**,**h**), respectively.

Figure 2 shows the SEM and 3D micrographs of the Kapton substrate and the PHPS coating surface before and after AO exposure. The pristine Kapton film surface was smooth and homogenous. The as-prepared PHPS coating was compact. Their 3D morphologies indicated that the roughness of the Kapton even reduced after being coated by PHPS coating. The eroded Kapton film surface exhibited a carpet-like structure (Figure 2c) [45] and the roughness of Kapton reached as much as 1.5 μm, indicating that it suffers from severe AO erosion. On the other hand, PHPS coatings after AO exposure exhibited quite a different morphology. Only minor defects and micro-cracks appeared in local areas after AO exposure, while most areas of the coating maintained their integrity. Moreover, the roughness of the PHPS coating became smaller after AO exposure (Figure 2h). Considering all the above, the PHPS coating exhibits sufficient resistance to AO erosion corrosion.

**Figure 3:**
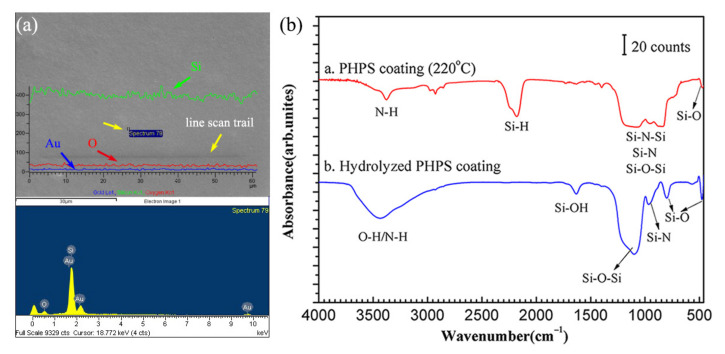
Chemical composition of the PHPS coating tested by (**a**) EDS line scan and point scan and (**b**) FTIR.

Figure 3a shows the EDS results of the PHPS coating. Prior to SEM investigation, the sample was coated with a thin layer of Au to ensure sufficient conductivity. Hence, Au was detected on the coating surface. The results obtained in the line-scan mode and point-scan mode reveal that the surface is composed of Si, O, and C, and the concentration of Si was significantly higher than that of other elements.

The PHPS coating was prepared on Kapton substrate according to the method as described in Section 2.1 and annealed at 220 °C. After annealing, one of the samples was directly tested by infrared spectrometer while another one was first dipped in water for 5 min, dried in air, then tested by infrared spectrometer. Their IR spectra are shown as ‘a’ and ‘b’ in Figure 3b, respectively. In both IR spectra, the absorbance bands at ~3385 and 2188 cm^−1^ correspond to the N-H and Si-H stretching vibrations, respectively. The absorbance characteristic of Si-N-Si bonds observed in the range of 830–1202 cm^−1^. Further, the bands located at 476 cm^−1^ correspond to the Si-O bonds. The spectra reveal that, firstly, before hydrolysis, the PHPS coating is composed of N-H, Si-H, Si-N-Si, Si-N, Si-O-Si, and Si-O. Moreover, there are abundant Si-H bonds in the PHPS coating. Then, after hydrolysis, the absorbance bands of Si-H bonds disappear completely, indicating that Si-H is decomposed during hydrolysis. Meanwhile, a strong absorbance band corresponding to Si-O-Si bonds emerged at 1103 cm^−1^. At the same time, the intensified Si-O absorbance bands at 476 cm^−1^ and 801 cm^−1^ indicate that the hydrolysis leads to the removal of N atoms from the coating and the incorporation of O atoms into the coating. The abundant Si-H bonds in PHPS are well known to react strongly with the hydroxyl groups in water. The curing of the PHPS coating in water at low temperatures led to the efficient conversion of Si-H to Si-O-Si linkages. Consequently, a high concentration of Si-O-Si cross-links is formed in the hydrolysed coating.

### 3.2. Adhesion Property of the PHPS Coating

**Figure 4:**
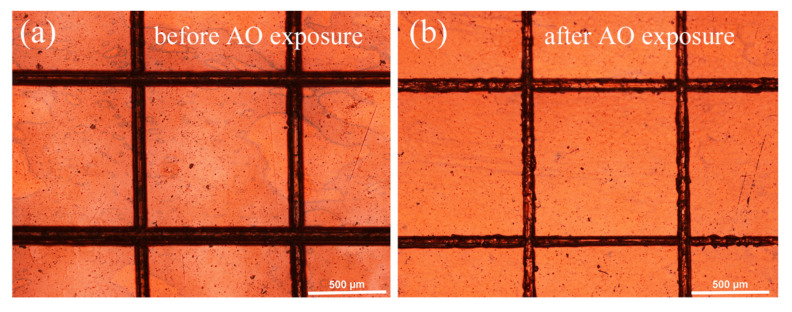
OM images of the coating after the cross-cut test (**a**) before and (**b**) after AO exposure.

The adhesion strength of the as-prepared PHPS coating was determined by the cross-cut test according to the International Standard ISO 2409. Figure 4 shows the optical microscope photographs of the coated Kapton after the cross-cut test before and after AO exposure. The photographs show that the cut edges are neat and tidy, which display that the flaking of the PHPS coating before and after AO exposure did not occur. According to classification methods described in ISO 2409, this result demonstrates that the adhesion strength of the PHPS coating to the Kapton substrate is excellent.

### 3.3. AO and AO + VUV Erosion

**Figure 5:**
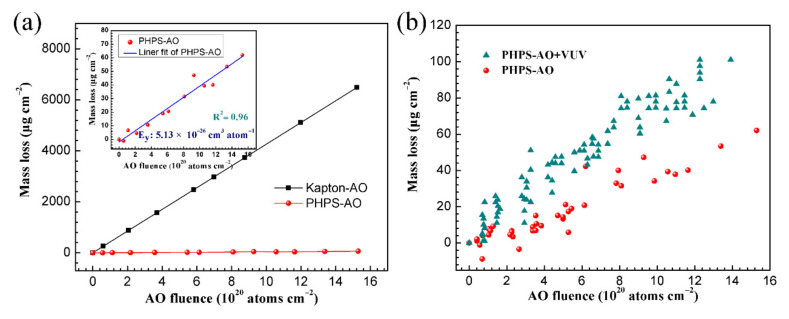
Dependence of the mass loss on the AO fluence for (**a**) prinstin and PHPS coated Kapton, that of the PHPS coated Kapton detailed in the inset figure and (**b**) PHPS coated Kapton after AO and AO + VUV exposure (Total fluence of AO exposure: 1.5 × 10^21^ atoms cm^−2^, VUV intensity: 9.56 μW cm^−2^).

The AO and AO + VUV erosion tests were conducted herein. Figure 5a shows the mass loss and erosion yield of the pristine and the coated Kapton. The pristine Kapton film was used as a reference, whose erosion yield is known as 3 × 10^−24^ cm^3^ atom^−1^. After being coated with PHPS, the mass loss of Kapton significantly decreased from 6.5 mg cm^−2^ to 0.062 mg cm^−2^ with a total AO exposure fluence of 1.5 × 10^21^ atoms cm^−2^ (Figure 5). The erosion yield of the PHPS coating was determined to be 5.13 × 10^−26^ cm^3^ atom^−1^ according to Equation (1), which was about two orders of magnitude less than that of pristine Kapton. According to the AO fluence on the serious erosion direction of the Hubble Space Telescope (HST) operated in LEO (about 1.2 × 10^21^ atoms cm^−^^2^), the survival time of the 1 μm thick PHPS coating in space environment was predicted to be about 48 years. Thus, the PHPS coating could considerably increase the service life of the polymers applied to the exterior of the spacecraft. In addition, the AO + VUV resistance performance of the PHPS coating was investigated. Figure 5b shows the dependence of the mass loss on the AO fluence of the PHPS coating. The result showed that with the participation of VUV irradiation, the mass loss of PHPS coated Kapton increased. However, the mass loss of the coating was just 0.12 mg cm^−2^ under a large AO flux of 2 × 10^16^ atoms cm^−2^ s^−1^ and an intense VUV intensity of 9.56 μW cm^−2^ for 21 h. Such a low mass loss indicates that the PHPS coating demonstrates good AO + VUV resistance.

### 3.4. XPS Analysis Results

**Figure 6:**
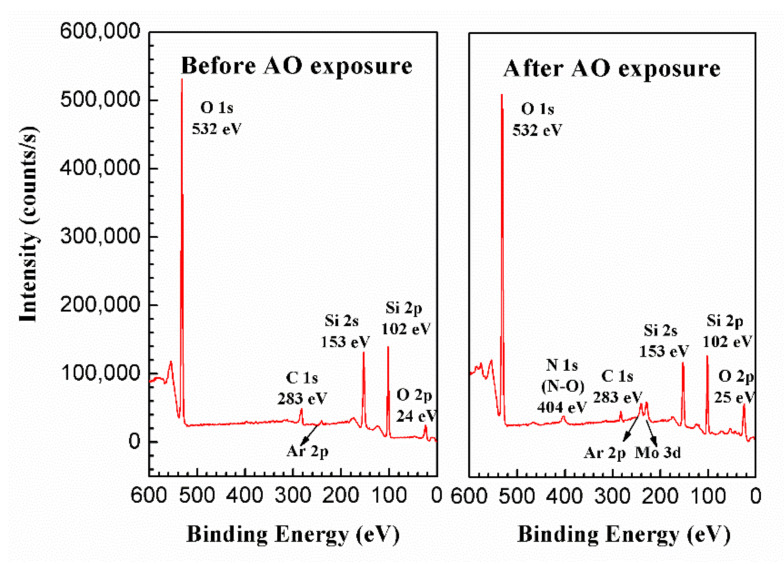
Survey spectra of the etched PHPS coating surface before and after AO exposure.

To reveal the AO resistance of the PHPS coating, the elemental composition and lateral and depth distribution of elements of the coating were investigated by XPS. Figure 6 shows the survey scan of the PHPS coating before and after AO exposure. To prevent the effect of the contamination layer on the sample, both samples were etched by Ar^+^ for 30 s before testing. The survey spectra reveal that the coating surface was simply and clearly composed of O, Si, C, and N elements. After AO exposure, the N 1s emerged as N-O. Peaks located at binding energies of 230 eV and 241 eV corresponded to Mo 3d5/2 and Ar 2p3/2, respectively. These were contaminations induced by the Mo plate (neutralizer in the AO erosion facility) and Ar-ion etching.

**Figure 7:**
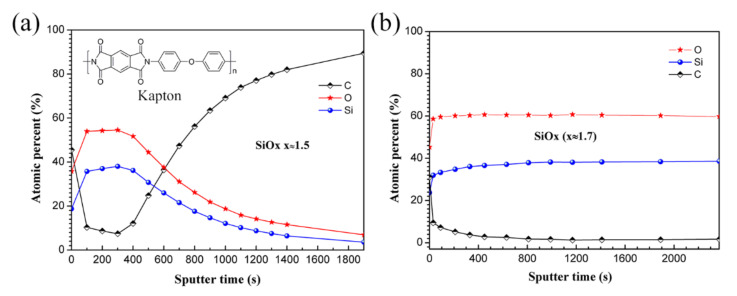
Depth profiles of C, O, Si elements in the (**a**) as-prepared and (**b**) AO-exposed PHPS coating (Total fluence of AO exposure: 1.5 × 10^21^ atoms cm^−2^).

The depth profile of each element can disclose the inner composition evolution of the PHPS coating before and after AO exposure. The coating surfaces were continuously etched by Ar^+^ built into the XPS, and the high-resolution spectra of each element were recorded every 100 s (Figure 7). The results reveal that after the first 100 s of etching, the contamination on both samples was totally removed. The main composition of the as-prepared PHPS coating includes Si and O, and the O/Si atomic ratio is about 1.5. After etching for 400 s, the concentrations of Si and O proportionally decreased, while that of C increased. Then, the concentration of C surpassed those of Si and O after etching for 600 s. Finally, the signals of Si and O nearly disappeared, and C was the only observed element. However, for the AO-exposed PHPS coating, the elemental composition was stable during the whole Ar^+^ etching process. The main composition of the coating was Si and O, with an O/Si atomic ratio of about 1.7, which remained unchanged during the 2370-s etching process.

Theoretically, the etching time could be used to evaluate the thickness of the material, because the etching time is directly proportional to the depth. Comparing the depth profiles of the as-prepared and AO-exposed PHPS coating, we found that the SiO_x_ layer on top of the AO-exposed PHPS coating might be thicker than that of the as-prepared PHPS coating. However, according to the law of conservation of mass, their thickness difference could not be this much. Considering that the etching rate is not only related with the energy of Ar^+^, but also related with the stiffness of the materials, we think the AO-exposed PHPS coating was just much more durable than the as-prepared PHPS coating under the same Ar^+^ etching process. According to this, we proposed that the Ar etching durability of the PHPS coating was strengthened by AO during the AO exposure. This deduction was in correspondence with the increase of the O/Si atomic ratio. At last, it is worth mentioning that the depth profiles were just used to reveal the element depth distribution and could not be used to evaluate the thickness of the coating in this work.

**Figure 8:**
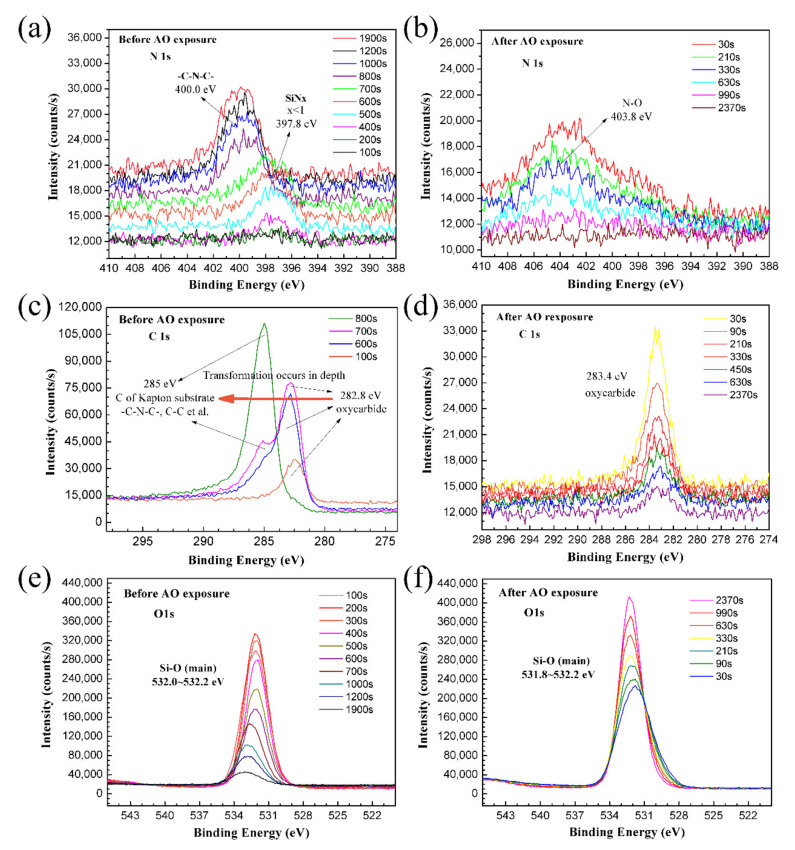
High-resolution XPS depth profiles of N 1s, C 1s and O 1s in the (**a**,**c**,**e**) as-prepared and (**b**,**d**,**f**) AO-exposed PHPS coating (Total fluence of AO exposure: 1.5 × 10^21^ atoms cm^−2^).

High-resolution narrow scans of the as-prepared and the AO-exposed PHPS coating were also detected, and the spectra are shown in Figure 8. For the as-prepared PHPS coating, the peak location of N 1s and C1s shifted during the etching. On the contrary, all peaks of the AO-exposed PHPS coating almost appeared at a fixed location during the etching and just their intensity changed. Figure 8a,b shows a set of N 1s spectra, which were composed of the N 1s spectrum obtained every 100 s from 30 s up to the end of the etching process. During the first 700 s of etching, only one peak located at ~397.8 eV was observed, corresponding to SiN_x_ (x < 1) (Figure 8a). This peak might be caused by the incomplete hydrolysis of Si-N in PHPS. The intensities of this peak in inner layers were stronger than those in the outer layers. When the etching progresses to 800 s, the peak located at 397.8 eV disappeared. At the same time, another peak emerged at 400.0 eV whose peak area increased with the etching time. The peak corresponding to the C-N-C in imide [44] was attributed to the Kapton substrate, whose molecular structure is shown in Figure 7a. For the AO-exposed PHPS coating, all of the peaks appeared at 403.8 eV during the etching process, which was attributed to N-O. This peak mainly resulted from the oxidation of residual Si-N in the incompletely hydrolyzed PHPS coating. However, the signal was weak, indicating that the amount of NO_x_ on the coating surface was little.

Figure 8c shows the chemical state transition of C on the as-prepared PHPS coating surface. After etching for 100 s, the C 1 s peak was observed at 282.8 eV, which corresponded to oxycarbide [46]. When etching for 600 s, the shape of the C 1s peak located at 282.8 eV changed. A bulge emerged at the higher-binding energy side of the oxycarbide peak. After etching for 700 s, an independent peak appeared at 285.0 eV, which corresponded to the organic C skeletons of the Kapton substrate. After etching for 800 s, just one sharp and strong peak was observed at 285.0 eV. According to the results, the emergent of peak 285.0 eV and the disappearance of peak 282.8 eV indicated that the chemical state transition process of C complete. For the AO-exposed PHPS coating, the locations of C 1s peaks were fixed at 283.4 eV during etching, which corresponded to oxycarbide. Compared with the C 1s spectra of the as-prepared PHPS coating, the amount of oxycarbide on the AO-exposed coating surface was less, and the peaks faded away with the etching time (Figure 8d).

The O 1s spectra of the as-prepared and the AO-exposed samples were all centred at 531.8–532.2 eV. Combined with the previously obtained result, the peak corresponded to Si-O. The peak intensity and relative change trends were different between the as-prepared and AO-exposed PHPS coating during etching (Figure 8e,f). For the as-prepared PHPS coating, the intensity of O 1s peak decreased with the etching time. Notably, after etching for 500 s, the intensity dramatically decreased. On the contrary, for the AO-exposed PHPS coating, the intensity of the O 1s peak strengthened with the etching time. This phenomenon was in correspondence with the hypothesis we proposed previously.

**Figure 9:**
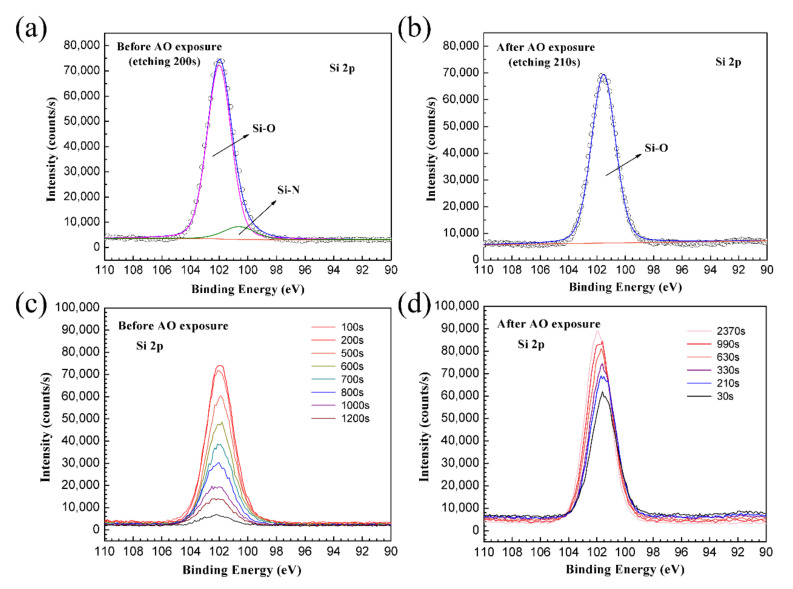
Deconvolution of Si 2p for (**a**) as-prepared and (**b**) AO-exposed PHPS coating, and high-resolution Si 2p XPS depth profiles of (**c**) as-prepared and (**d**) AO-exposed PHPS coating (Total fluence of AO exposure: 1.5 × 10^21^ atoms cm^−2^).

Si was another main element of the coating. Two peaks were observed in the Si 2p spectrum of the as-prepared coating as shown in Figure 9a. One peak at 102.0 eV and the other peak at 100.6 eV corresponded to SiO_x_ and Si-N, respectively. These peaks originated from the incomplete hydrolysis of the PHPS coating. Only one peak appeared in the Si 2p spectrum of the AO-exposed PHPS coating (Figure 9b). The peak was situated at ~101.6 eV and corresponded to SiO_x_. The Si 2p peak intensity of the as-prepared PHPS coating dramatically decreased during etching progress (Figure 9c). On the contrary, that of the AO-exposed PHPS coating just slightly decreased after etching for 2370 s (Figure 9d). According to all of the XPS results, we concluded that AO exposure induced the increase of the O/Si atomic ratio of the SiO_x_ layer on top surface of the PHPS coating, which strengthened the durability of the coating.

### 3.5. AO Resistance Mechanism of the PHPS Coating

**Figure 10:**
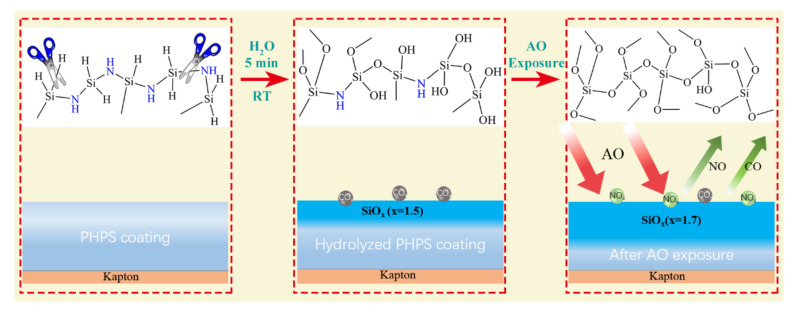
Schematic of the chemical reactions and the composition evolution of the PHPS coating during the hydrolysis and AO exposure.

The surface elemental composition of the PHPS coating is crucial for its AO resistance performance. Two processes were investigated in this study: PHPS hydrolytic process and AO exposure process. A large number of Si-H groups of PHPS could react strongly with hydroxyl groups of water. The Si-N groups also undergo hydrolysis under suitable humidity and temperature. By the hydrolysis of PHPS with water, silanol groups are formed as intermediates. Finally, condensation and cross-linking reactions lead to the formation of SiO_x_. According to the report [30,42], the hydrolysis rate is affected by various factors, e.g., humidity, temperature, and catalyst. Among these factors, humidity is the key factor [42]. After 180 min at 50 °C in ‘dry’ air, the conversion of Si-H groups is ~15% compared to ~55% in the presence of moisture [30]. In this study, the PHPS coating hydrolyzed in water for 5 min at room temperature; Partial hydrolysis of the PHPS coating happened, which was confirmed by FTIR and XPS test results. This process constructed a composition gradient in the PHPS coating. The formed intrinsic SiO_x_ layer on the top surface of the coating elevated its AO erosion resistance, and the originally formed covalent bond between the PHPS and the hydroxyl modified Kapton in the interface maintained the PHPS coating bond with the Kapton substrate. XPS results demonstrated the entire evolution process of elements with the depth of the PHPS coating, especially Si-O evolution. Figure 10 shows the schematic of the chemical reactions and the gradient surface of the as-prepared PHPS coating.

An interesting phenomenon in this study is that the coating exhibited better Ar^+^ etching resistance after AO exposure. The sample thickness is well known to be inversely proportional to the Ar^+^ etching time in general. However, the coating after AO exposure could bear much longer Ar^+^ etching than the as-prepared PHPS coating, i.e., even after etching for 2370 s, the substrate signal was not detected, indicating that the coating was not damaged. Therefore, the durability of the PHPS coating is concluded to be significantly lifted after AO exposure. The erosion resistance ability of the coating is decided by the PHPS-to-SiO_x_ conversion degree. As the PHPS-to-SiO_x_ conversion proceeded, the O/Si atomic ratio increased. Its value was ~2 when the PHPS-to-SiO_x_ conversion was completed [42]. In this study, the O/Si atomic ratio of the coating increased from 1.5 to 1.7 after AO exposure. According to the O/Si atomic ratio of the coating and the peak area of C 1s and N 1s, it was proposed that, on the one hand, AO with kinetic energy led to the erosion of the coating surface, and on the other hand, AO removed the contamination and oxidized the PHPS coating further. These results confirmed that a layer of stiff SiO_x_ formed immediately on the as-prepared PHPS coating surface after AO exposure, which is the key reason for the efficient AO erosion resistance of the PHPS coating (Figure 10).

## 4. Conclusions

To protect polymers from AO and VUV damage in the low Earth orbit, a PHPS-derived SiO_x_ coating was prepared. The bonding and AO/VUV erosion-resistance effect and relative mechanism of the PHPS-derived SiO_x_ coating were investigated. They were mainly investigated by analysing the element distribution in the depth direction of the coating. The results of our analysis showed that a gradient distribution of the composition emerged in the PHPS coating on Kapton, which means the PHPS coating was incompletely hydrolyzed. At the bottom of the incompletely hydrolyzed coating, PHPS still covalently bonded with the modified Kapton by forming hydroxyl, and on the top surface of the PHPS coating a layer of SiO_x_ was formed during hydrolysis, which led to the good bonding property and AO erosion-resistance of the PHPS coating. Moreover, the elemental composition and lateral and depth distribution of elements of the PHPS coating displayed that the O/Si atomic ratio increased from 1.5 to 1.7 after the coating was exposed in AO. This could be the reason why the PHPS coating exhibited excellent AO erosion-resistance. According to these results, an AO resistance mechanism model of the PHPS coating is constructed. In addition, the AO + VUV resistance experiment reveals that the mass loss of the PHPS coating was still less than 0.12 mg cm^−2^ under the large AO fluence of 1.4 × 10^21^ atoms cm^−2^ and intense VUV intensity exposure condition, which indicated that the PHPS coating exhibits good AO + VUV resistance.

## Data Availability

The raw/processed data required to reproduce these findings cannot be shared at this time due to technical or time limitations.
